# Downregulation of 26S proteasome catalytic activity promotes epithelial-mesenchymal transition

**DOI:** 10.18632/oncotarget.7596

**Published:** 2016-02-22

**Authors:** Asoka Banno, Daniel A. Garcia, Eric D. van Baarsel, Patrick J. Metz, Kathleen Fisch, Christella E. Widjaja, Stephanie H. Kim, Justine Lopez, Aaron N. Chang, Paul P. Geurink, Bogdan I. Florea, Hermen S. Overkleeft, Huib Ovaa, Jack D. Bui, Jing Yang, John T. Chang

**Affiliations:** ^1^ Department of Medicine, University of California San Diego, La Jolla, CA, USA; ^2^ Center for Computational Biology and Bioinformatics, Institute for Genomic Medicine, University of California San Diego, La Jolla, CA, USA; ^3^ Division of Chemical Biology, Leiden Institute of Chemistry, Leiden University, Gorlaeus Laboratories, Leiden, The Netherlands; ^4^ Division of Cell Biology, The Netherlands Cancer Institute, Amsterdam, The Netherlands; ^5^ Department of Pathology, University of California San Diego, La Jolla, CA, USA; ^6^ Department of Pharmacology, University of California San Diego, La Jolla, CA, USA; ^7^ Department of Pediatrics, University of California San Diego, La Jolla, CA, USA; ^8^ Moores Cancer Center, University of California San Diego, La Jolla, CA, USA

**Keywords:** EMT, proteasome, TGF-beta, cancer stem cells

## Abstract

The epithelial-mesenchymal transition (EMT) endows carcinoma cells with phenotypic plasticity that can facilitate the formation of cancer stem cells (CSCs) and contribute to the metastatic cascade. While there is substantial support for the role of EMT in driving cancer cell dissemination, less is known about the intracellular molecular mechanisms that govern formation of CSCs via EMT. Here we show that β2 and β5 proteasome subunit activity is downregulated during EMT in immortalized human mammary epithelial cells. Moreover, selective proteasome inhibition enabled mammary epithelial cells to acquire certain morphologic and functional characteristics reminiscent of cancer stem cells, including CD44 expression, self-renewal, and tumor formation. Transcriptomic analyses suggested that proteasome-inhibited cells share gene expression signatures with cells that have undergone EMT, in part, through modulation of the TGF-β signaling pathway. These findings suggest that selective downregulation of proteasome activity in mammary epithelial cells can initiate the EMT program and acquisition of a cancer stem cell-like phenotype. As proteasome inhibitors become increasingly used in cancer treatment, our findings highlight a potential risk of these therapeutic strategies and suggest a possible mechanism by which carcinoma cells may escape from proteasome inhibitor-based therapy.

## INTRODUCTION

Cancer is one of the leading causes of death in the United States, and up to 90% of cancer-associated mortality can be attributed to therapy-resistant metastatic disease [1]. During the metastatic cascade, tumor cells gain the capacity to invade locally and disseminate into the vasculature. However, not all cells that enter the vasculature go on to colonize distant sites. Only a small subset of invading tumor cells acquire characteristics of cancer stem cells (CSCs) needed to establish macrometastases, namely self-renewal capacity, proliferative potential, and chemoresistance [2]. There is increasing evidence to support the involvement of CSCs in multiple types of hematologic and solid tumors, including breast, brain, prostate, colon, liver, and pancreatic, among others [3]. Although the ontogeny of CSCs is incompletely understood, a developmental process known as the epithelial-mesenchymal transition (EMT) has been shown to promote the development of cells with CSC properties [4-8]. Previous studies have identified the transcription factors TWIST1, SNAI1, and ZEB1 as key inducers of EMT, metastasis, and the CSC phenotype [3, 6, 8-10]. Thus, identifying the factors that regulate EMT is highly relevant to cancer therapy as these stimuli can be targeted to block metastasis, and potentially CSC formation, in carcinomas.

Recent evidence suggests that CSCs may exhibit decreased proteasome activity [11-15]. The 26S proteasome is comprised of a 20S core complex that contains β1, β2, and β5 catalytic subunits that contain caspase-like, trypsin-like, and chymotrypsin-like proteolytic sites, respectively [16, 17]. The observation of decreased proteasome activity in CSCs led us to hypothesize that downregulation of proteasome activity might be causally related to the acquisition of the CSC phenotype, *via* EMT.

Here we show that immortalized human mammary epithelial (HMLE) cells and MCF10A cells, both well-established model systems for EMT [6], decrease their proteasome activity as they undergo EMT. Strikingly, we observed that selective inhibition of β2 or β5 subunit proteasome activity was sufficient to induce HMLE and MCF10A cells to acquire key morphologic and functional characteristics of the EMT. Transcriptomic analyses suggested that proteasome-inhibited cells share gene expression signatures with cells that had undergone EMT, in part, through modulation of the TGF-β signaling pathway. Taken together, these data suggest that downregulation of proteasome activity in breast cancer cells can initiate the EMT program, thereby conferring upon these cells key attributes of CSCs.

## RESULTS

### Downregulation of proteasome activity is associated with EMT

**Figure 1:**
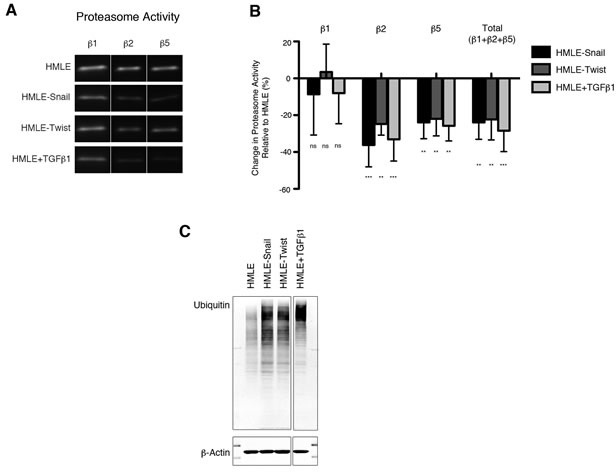
Downregulation of proteasome activity is associated with EMT **A.** β1, β2, and β5 subunit proteasome activity in HMLE, HMLE-Snail, HMLE-Twist, and HMLE+TGF-β1 were measured by in-gel proteasome activity assay. Representative images of the SDS-PAGE gels are shown. Vertical spaces inserted between lanes indicate removal of intervening, irrelevant samples. All the samples were run on the same gel and imaged in a single scan. **B.** Quantification of β1, β2, and β5 subunit activity as well as total catalytic activity (the sum of the three subunits) presented as percent change relative to HMLE. Error bars indicate standard error of the mean (SEM) (*n* ≥ 3). **C.** Immunoblot of whole cell lysates from HMLE cells using an anti-ubiquitin antibody, representative of 3 independent experiments. β-actin served as a loading control. Vertical spaces inserted between lanes indicate removal of intervening, irrelevant samples. All the samples were run on the same gel, transferred and blotted together, and imaged in a single scan.

We first sought to determine whether cells undergoing EMT alter their levels of proteasome activity. We utilized HMLE cells in which EMT can be induced by stable overexpression of *SNAI1* or *TWIST1*, or by treatment with TGF-β1 (hereafter referred to as HMLE-Snail, HMLE-Twist, or HMLE+TGF-β1, respectively), as previously described [6, 18-22]. We determined proteasome activity by using subunit-specific probes that bind irreversibly to the β1, β2, or β5 proteasome catalytic subunits (Supplementary Figure S1) [23-25]. We observed that cells that had undergone EMT exhibited a 25-30% reduction in β2 and β5, but not β1, subunit activity compared to cells that had not undergone EMT (Figure 1A, 1B). This is likely due to a reduction in specific proteasome activity, since total protein and mRNA expression of these subunits remained unaffected by EMT induction (Supplementary Figure S2A and S2B). In support of this finding, we observed that cells that had undergone EMT also exhibited an accumulation of ubiquitinated proteins, compared to their epithelial parental cells (Figure 1C). Taken together, these data suggest that HMLE cells decrease specific proteasome catalytic activities - not proteasome amounts - during EMT.

### Selective inhibition of proteasome activity induces the EMT phenotype

**Figure 2:**
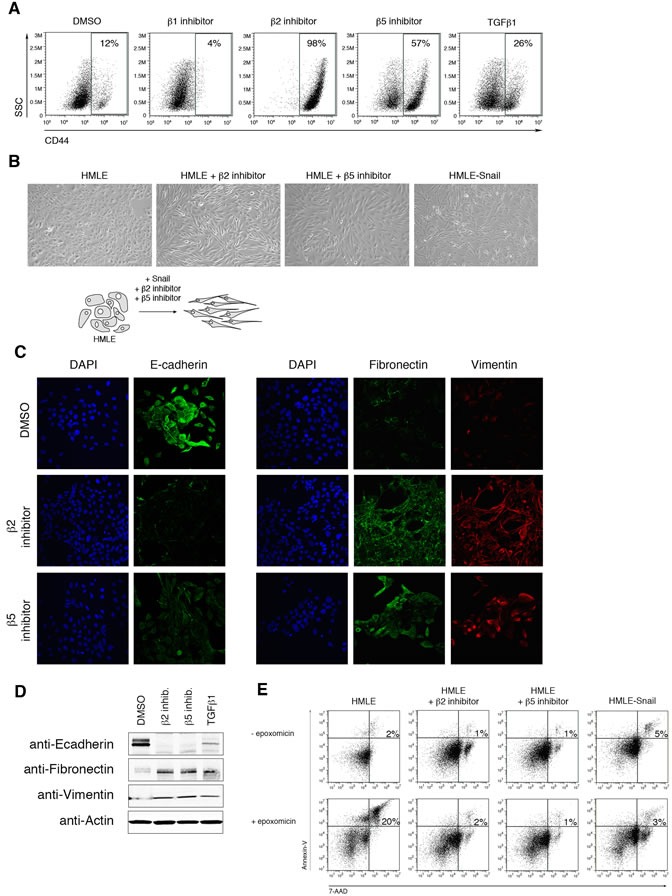
Selective inhibition of proteasome activity induces an EMT phenotype **A.** Flow cytometry analysis of CD44 surface expression and side scatter (SSC) after 14 days of treatment with DMSO or β1, β2, or β5 subunit inhibitor. Percentage of CD44^high^ cells within the live population is indicated. Representative result of three independent experiments is shown. **B.** Representative brightfield images of HMLE, HMLE+β2 inhibitor, HMLE+β5 inhibitor, and HMLE-Snail after 14 days of treatment. All the images were taken at 10X magnification. Schematic diagram depicts the change in cell morphology during EMT. **C.** Confocal microscopy of E-cadherin (left panel; green), fibronectin (right panel; green), or vimentin (red) in HMLE cells treated with β2 subunit inhibitor or β5 subunit inhibitor for 14 days. Images were taken at 40X magnification. **D.** Immunoblot of whole cell lysates from HMLE, HMLE+β2 inhibitor, HMLE+β5 inhibitor, or HMLE+TGF-β1 using anti-E-cadherin, anti-fibronectin, and anti-vimentin antibodies, representative of 3 independent experiments. β-actin served as a loading control. **E.** Flow cytometric analysis of 7-AAD and Annexin-V expression in HMLE, HMLE+β2 inhibitor, HMLE+β5 inhibitor, and HMLE-Snail with or without 1 day of epoxomicin treatment. Percentage of 7-AAD^+^/AnnexinV^+^ cells is indicated. Representative result of three independent experiments is shown.

To investigate whether the reduction in proteasome activity is mechanistically linked to the process of EMT, we treated HMLE cells with selective β1, β2, or β5 proteasome subunit inhibitors (Supplementary Figure S1) [25-27]. We then assessed the cell surface expression of CD44 by HMLE cells after 14 days of treatment. High expression of CD44 has been associated with human breast cancer stem cells [28, 29] as well as with HMLE cells that have undergone EMT [6]. Strikingly, 98% of cells treated with β2 subunit inhibitor and 57% of those treated with β5 subunit inhibitor expressed high levels of CD44, compared to 12% of DMSO-treated cells (Figure 2A). By contrast, cells treated with the β1 subunit inhibitor expressed low levels of CD44 (Figure 2A), consistent with the lack of change in β1 subunit proteasome activity within cells that had undergone EMT (Figure 1A, 1B). To exclude the possibility that the increase of the CD44^high^ population was due to selective outgrowth of CD44^high^ cells, HMLE cells were first FACS sort-purified for low expression of CD44, then treated with selective proteasome inhibitors (Supplementary Figure S3A). We found that CD44^low^ cells treated with proteasome inhibitors gave rise to CD44^high^ cells after 14 days of treatment (Supplementary Figure S3B), demonstrating that these cells arose directly from CD44^low^ cells.

CD44^high^ cells that emerged after treatment with selective β2 or β5 subunit inhibitors lost their cobblestone-like appearance and acquired the fibroblast-like morphology characteristic of mesenchymal cells (Figure 2B). Moreover, cells treated with selective proteasome inhibitors decreased their expression of epithelial marker E-cadherin and increased their expression of mesenchymal markers fibronectin and vimentin, as shown by immunofluorescence and immunoblot analyses (Figure 2C, 2D). Together, these results suggest that selective β2 or β5 subunit inhibition induces HMLE cells to acquire an EMT phenotype.

In addition to exhibiting mesenchymal characteristics, we found that cells with lower levels of proteasome activity exhibited decreased apoptosis, in comparison to parental HMLE cells, when treated with the pan-proteasome inhibitor epoxomicin (Figure 2E). These results suggest that the reduced level of proteasome activity associated with EMT confers increased resistance to the cytotoxic effects of pan-proteasome inhibition. Furthermore, these results are consistent with prior observations that CSCs may be more resistant to proteasome inhibitors [14].

We found that a second non-tumorigenic human mammary epithelial cell line, MCF10A, also downregulated β2 and β5 subunit activity while undergoing EMT in response to TGF-β1 (Supplementary Figure S4A). MCF10A cells have been previously shown to decrease their expression of CD24 as they undergo EMT [7]. We observed a decrease in CD24 expression in MCF10A cells treated with selective β2 or β5 subunit inhibitors, suggesting that these cells had undergone EMT (Supplementary Figure S4B). In addition, MCF10A cells treated with β2 or β5 subunit inhibitors exhibited a fibroblast-like morphology, decreased expression of epithelial markers, and increased expression of mesenchymal markers at the protein and mRNA levels (Supplementary Figure S4C-F). Together, these results suggest that induction of the EMT phenotype as a result of selective β2 or β5 inhibition of proteasome activity may be a generalizable phenomenon.

### Cells treated with selective proteasome inhibitors acquire the ability to self-renew

**Figure 3:**
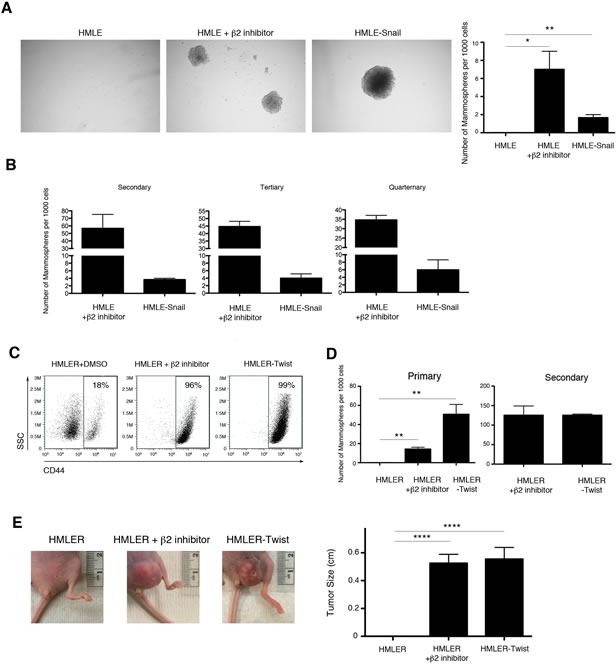
Selective inhibition of proteasome activity endows HMLE cells with self-renewal ability *in vitro* and tumor-initiating capacity *in vivo* A. Quantification of primary mammospheres per 1000 seeded cells formed by HMLE, HMLE+β2 inhibitor, or HMLE-Snail. Error bars indicate SEM (*n* ≥ 3). **B.** Serial passage of mammospheres. Quantification is presented in the bar graph as the number of mammospheres formed per 1000 seeded cells. Error bars indicate SEM (*n* 3). **C.** Flow cytometric analysis of CD44 expression by HMLER-Twist, β2 subunit inhibitor-treated or DMSO-treated HMLER cells. Percentage of CD44^high^ cells within the live population is indicated. Representative result of three independent experiments is shown. **D.** Quantification of primary and secondary mammospheres formed by HMLER, HMLER+β2 inhibitor, or HMLER-Twist, presented in the bar graph as the number of mammospheres formed per 1000 seeded cells. Error bars indicate SEM (*n* ≥ 3). **E.** Primary tumor formation in immunodeficient mice 2 months after injection with HMLER, HMLER+β2 inhibitor, or HMLER-Twist cells. Error bars indicate SEM (*n* = 12 mice per group). Representative images of the tumors are also shown.

We next sought to confirm that the CD44^high^ cells that had arisen following treatment with proteasome inhibitors had indeed undergone EMT. We focused our studies on β2 subunit inhibition due to its more pronounced effect (Figure 2A). It has been previously demonstrated that HMLE cells that have undergone EMT exhibit an increased ability to self-renew, a characteristic typically associated with mammary epithelial stem cells [6, 30, 31]. We therefore tested the ability of HMLE cells treated with proteasome inhibitors to form mammospheres, a capability indicative of self-renewal activity. Indeed, β2 subunit inhibitor-treated HMLE cells acquired an enhanced capacity to form mammospheres compared to DMSO-treated cells, both in primary assays and during subsequent serial passages (Figure 3A, 3B). These results suggest that selective proteasome inhibition confers self-renewal capabilities to HMLE cells.

### Selective inhibition of proteasome activity endows HMLER cells with tumor-initiating capacity *in vivo*

We next wished to determine whether selective proteasome inhibition also endowed HMLE cells with the ability to initiate tumors, another property of CSCs. Accordingly, we used a tumor xenograft system in which HMLE cells constitutively expressing *RAS-V12H* oncogene and *TWIST1* (HMLER-Twist) renders them tumorigenic when injected into immunodeficient mice [32]. Similar to the behavior of proteasome-inhibited HMLE cells described above, HMLER cells treated with β2 subunit inhibitor acquired a CD44^high^ phenotype and the ability to form mammospheres (Figure 3C, 3D). To test the tumorigenic potential of these cells, we injected HMLER, HMLER-Twist, or β2 subunit inhibitor-treated HMLER cells into immunodeficient mice. 92% of mice injected with HMLER-Twist or β2 inhibitor-treated HMLER cells developed tumors with a mean size of 0.5 cm; in contrast, mice injected with DMSO-treated HMLER cells did not develop tumors (Figure 3E). Taken together, these results suggest that downregulation of proteasome activity in HMLER cells can induce EMT and confer self-renewal and tumor-initiating capabilities, which are hallmarks of CSCs.

### Low proteasome subunit expression is associated with human breast cancer and decreased patient survival

**Figure 4:**
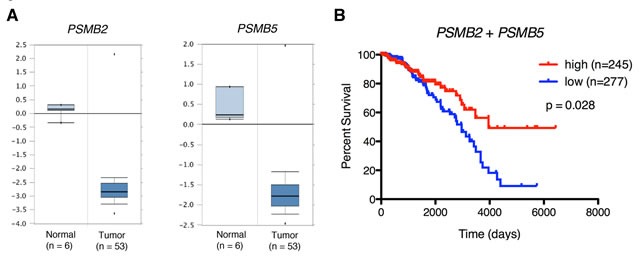
Low proteasome subunit expression is associated with breast tissue from patients with breast cancer and decreased patient survival **A.** *PSMB2* and *PSMB5* gene expression box plots from normal and tumor samples from the Finak dataset [47]. *P* < 0.01 for both comparisons. **B.** Kaplan-Meier survival curves for breast cancer patients stratified by intra-tumor expression (“high” *vs* “low”) of *PSMB2* and *PSMB5* mRNA.

To determine whether the link between reduced proteasome activity and tumorigenicity might apply to human patients with breast carcinoma, we analyzed publicly available gene expression data from the Oncomine Platform and The Cancer Genome Atlas. Intriguingly, we found that tumor samples from patients with invasive breast carcinoma exhibited significantly lower *PSMB2* and *PSMB5* proteasome subunit mRNA expression compared to samples derived from normal tissue (Figure 4A). Moreover, breast cancer patients with tumors exhibiting the lowest quartile of *PSMB2* and *PSMB5* combined mRNA expression showed reduced 5-year survival compared to patients with tumors in the highest quartile (Figure 4B). Together, these data suggest that low proteasome subunit expression may be useful as an indicator of poor prognosis for breast cancer patients.

### Selective inhibition of proteasome activity induces an EMT transcriptional program

**Figure 5:**
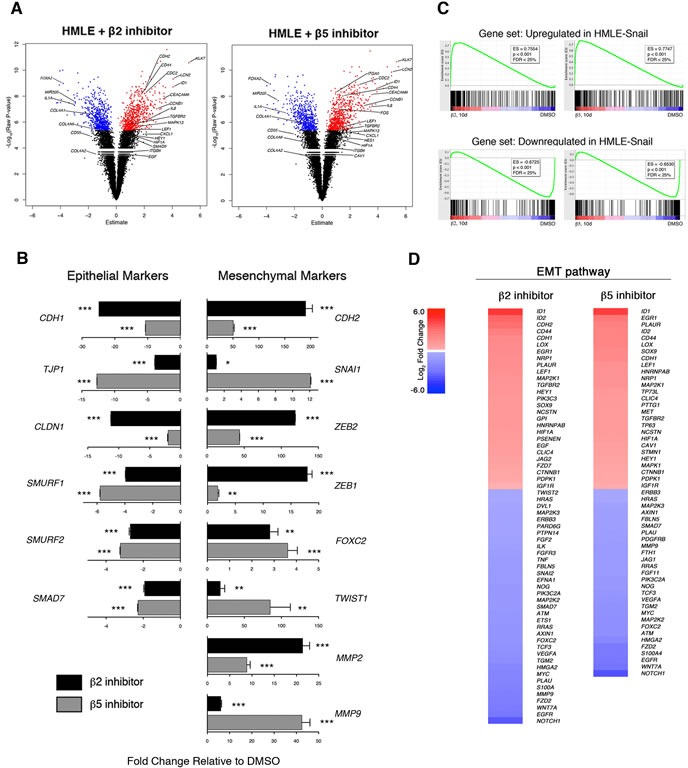
Selective inhibition of proteasome activity induces an EMT transcriptional program **A.** Volcano plots depicting differentially expressed genes in HMLE cells treated with β2 or β5 subunit inhibitors compared to DMSO-treated cells after 10 days. X-axis represents the Array Studio estimate and the y-axis represents the -Log_10_(Raw P-value). Significant differentially expressed genes are highlighted in red (upregulated) or in blue (downregulated). Selected genes of interest are indicated. **B.** mRNA levels of epithelial and mesenchymal markers in sorted CD44^high^ HMLE cells treated with β2 (black bars) or β5 inhibitor (grey bars). GAPDH was used as a reference gene. Data are shown as fold change relative to DMSO-treated HMLE. Error bars indicate SEM (*n* ≥ 3). **C.** GSEA plots. Enrichment score is visualized in green. ES, enrichment score. FDR, false discovery rate. **D.** Heatmaps depicting Log_2_ fold change of differentially expressed genes in the EMT pathway in HMLE cells treated with β2 or β5 subunit inhibitors compared to DMSO-treated cells. Gene list was generated with IPA.

To investigate the molecular mechanisms underlying proteasome inhibitor-induced EMT, we performed gene expression microarray analysis of DMSO-treated HMLE cells, cells treated with either β2 or β5 subunit inhibitors, and cells expressing Snail. 1,338 and 1,278 genes were differentially expressed in β2 and β5 subunit inhibitor-treated HMLE cells, respectively, compared to DMSO-treated cells. Among these differentially expressed genes were a number of transcripts that have been previously reported to be associated with EMT (Figure 5A). These included a number of upregulated mesenchymal markers *ID1*, *KLK7*, *LCN2*, and *CEACAM6* as well as downregulated epithelial markers *FOXA2*, *MIR205*, and *COL4A2* [9]. Moreover, key genes were validated by qPCR and we found that β2 and β5 inhibitor-treated HMLE cells exhibited increased expression of the EMT-associated transcription factors, *SNAI1, ZEB1/2*, and *TWIST1*, as well as decreased expression of epithelial markers *CDH1*, *TJP1*, and *CLDN1* (Figure 5B).

Gene set enrichment analysis (GSEA) revealed that transcripts upregulated or downregulated upon proteasome inhibitor treatment were significantly enriched within the set of genes upregulated or downregulated in HMLE-Snail, respectively (Figure 5C). These observations were confirmed by additional GSEA using datasets derived from the Molecular Signatures Database (Supplementary Table 1). Lastly, we applied Ingenuity Pathway Analysis (IPA) to the transcripts that were differentially expressed between β2 or β5 subunit inhibitor-treated HMLE cells and DMSO-treated cells. This analysis identified cellular movement, cell death and survival, cell growth and proliferation, and EMT as some of the most significantly enriched molecular and cellular functions across all samples (Table 1 and Figure 5D). Taken together, these analyses provide molecular evidence that β2 or β5 subunit inhibition induces HMLE cells to undergo EMT.

### Selective proteasome inhibitor induces an EMT transcriptional program by stabilizing the TGF-β1 signaling pathway

**Figure 6:**
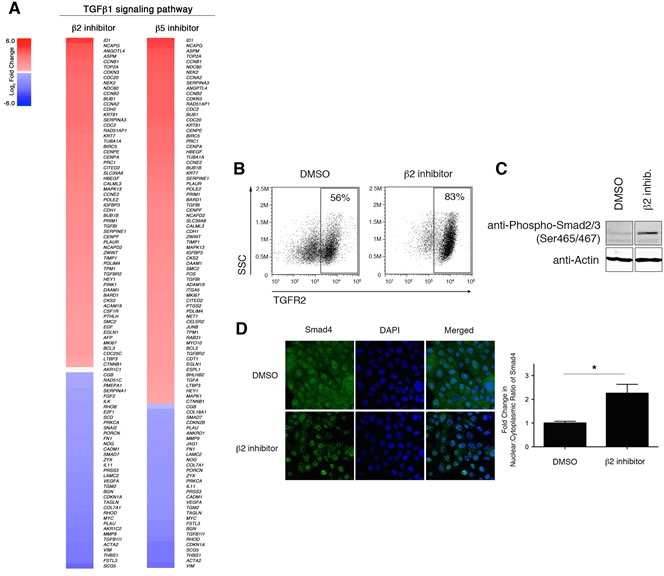
Downregulation of proteasome activity enhances TGF-β1 signaling A. Heatmaps depicting Log_2_ fold change of differentially expressed genes in the TGF-β1 signaling pathway in HMLE cells treated with β2 or β5 subunit inhibitors compared to DMSO-treated cells. **B.** Flow cytometric analysis of surface TGFR2 expression and SSC by HMLE cells treated for 1 day with DMSO or β2 subunit inhibitor. Percentage of TGFR2^+^ cells within the live population is indicated. Representative result of three independent experiments is shown. **C.** Immunoblot using anti-Phospho Smad2 (Ser465/467) antibody on whole cell lysates from DMSO- or beta2 inhibitor-treated HMLE cells for 24 hours representative of 3 independent experiments. β-actin served as a loading control. Vertical spaces inserted between lanes indicate removal of intervening, irrelevant samples. All the samples were ran on the same gel, transferred and blotted together, and imaged in a single scan. **D.** Confocal microscopy of Smad4 (green) and DNA (blue; stained with DAPI) in HMLE cells treated with β2 subunit inhibitor for 3 hours. Images were taken at 40X magnification. Bar graph presents fold change in nuclear/cytoplasmic ratio relative to control treatment. Error bars indicate SEM (*n* ≥ 3).

In addition to identifying a link between proteasome inhibition and the EMT pathway, IPA analysis also identified the TGF-β1 signaling pathway as a putative upstream regulator driving the observed transcriptional changes in proteasome-inhibited HMLE cells (Table 1 and Figure 6A). TGF-β1 signals through tetrameric TGF-β receptors 1 (TGFR1) and 2 (TGFR2) complexes, which phosphorylate SMAD2 (pSMAD2). SMAD4 combines with pSMAD2 to form multimeric SMAD-complexes that translocate into the nucleus and regulate transcription [9]. We hypothesized that reduced proteasome activity might result in increased stability of TGFR2 leading to increased TGF-β1 signaling. In support of this hypothesis, we observed increased cell surface expression of TGFR2 in HMLE cells treated with β2 subunit inhibitor (Figure 6B). Moreover, we found that treatment with β2 subunit inhibitor increased SMAD2 phosphorylation (Figure 6C) and increased SMAD4 nuclear localization by 2-fold (Figure 6D), compared to DMSO-treated cells. In line with these results, we also observed increased mRNA expression of SMAD target genes *SNAI1*, *ZEB1*/*2*, *MMP2*, and *MMP9*, as well as decreased expression of *SMAD7*, a negative regulator of TGF-β1 signaling (Figure 5B).

**Table 1:** IPA analysis of differentially expressed genes

		*p*-value		
Category	No. of molecules	HMLE-Snail	HMLE + β2 inhib.	HMLE + β5 inhib.
Cellular Movement	292	6.6 × 10^−21^	2.2 × 10^−11^	1.4 × 10^−11^
Cell Death & Survival	424	4.0 × 10^−20^	6.4 × 10^−16^	4.3 × 10^−15^
Cellular Growth & Proliferation	432	1.6 × 10^−18^	7.5 × 10^−15^	5.4 × 10^−14^
Cell Cycle	233	7.9 × 10^−16^	1.5 × 10^−10^	2.8 × 10^−13^
Epithelial-Mesenchymal Transition	24	4.0 × 10^−4^	7.7 × 10^−3^	n/a
Cell Cycle: G2/M DNA Damage Checkpoint Regulation	23	5.4 × 10^−9^	1.1 × 10^−6^	7.0 × 10^−7^
TGFβ1 Signaling	99	1.9 × 10^−23^	1.2 × 10^−20^	1.0 × 10^−22^

Transcripts differentially expressed in β2 or β5 inhibitor-treated HMLE cells are enriched in molecular and cellular functions involved in EMT.

**Figure 7:**
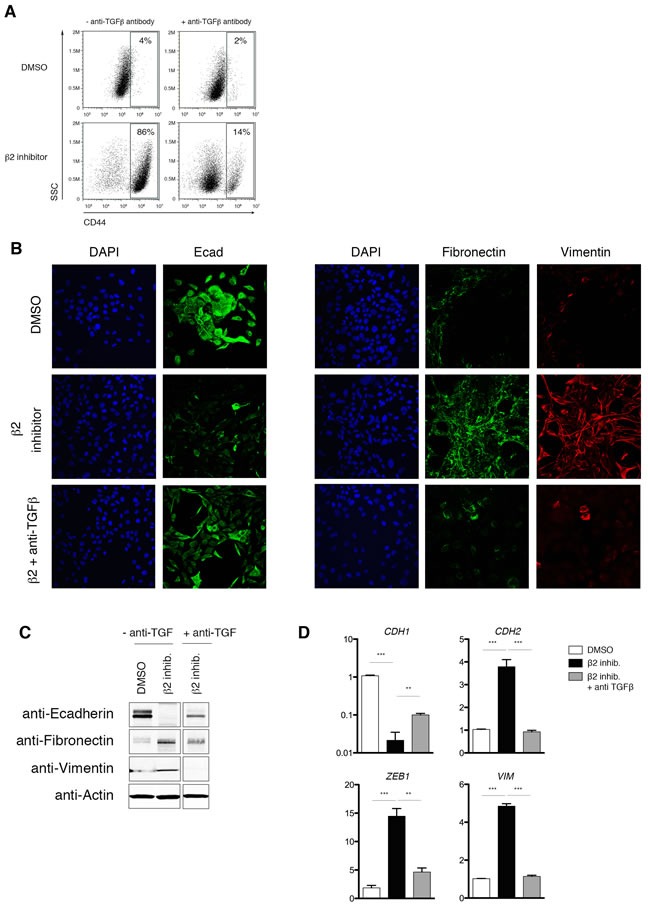
Proteasome inhibitor-induced EMT is dependent on TGF-β1 signaling **A.** Flow cytometric analysis of CD44 expression after 14 days of treatment with DMSO or β2 inhibitor, with or without neutralizing anti-TGF-β1 antibody. Percentage of CD44^high^ cells within the live population is indicated. Representative result of three independent experiments is shown. **B.** Confocal microscopy of E-cadherin (left panel; green), fibronectin (right panel; green), or vimentin (red) in HMLE cells, HMLE cells+β2 subunit inhibitor, or HMLE cells+β2 subunit inhibitor with anti-TGF-β1 antibody for 14 days. Images were taken at 40X magnification. **C.** Immunoblot of whole cell lysates from HMLE, HMLE+β2 inhibitor, or HMLE cells+β2 subunit inhibitor treated with anti-TGF-β1 antibody, using anti-E-cadherin, anti-fibronectin, and anti-vimentin antibodies. Images are representative of 3 independent experiments. β-Actin served as a loading control. Vertical spaces inserted between lanes indicate removal of intervening, irrelevant samples. All the samples were ran on the same gel, transferred and blotted together, and imaged in a single scan. **D.** mRNA levels of epithelial (*CDH1*) and mesenchymal markers (*CDH2*, *ZEB1*, and *VIM*) in HMLE or HMLE+β2 inhibitor treated with or without neutralizing anti-TGF-β1 antibody. GAPDH was used as a reference gene. Data are shown as fold change relative to DMSO-treated HMLE. Error bars indicate SEM (*n* ≥ 3).

To confirm that EMT induction in proteasome inhibitor-treated HMLE cells is due to TGF-β1 signaling, we repeated inhibitor treatment in the presence of an anti-TGF-β1 neutralizing antibody. We first validated the specificity of the neutralizing antibody in our system by showing that SMAD2 phosphorylation is blocked when HMLE cells are treated with TGF-β1 or proteasome inhibitors in the presence of the neutralizing antibody (Supplementary Figure S5A-B). Strikingly, we found that neutralizing anti-TGF-β1 antibodies prevented the β2 subunit inhibitor-induced increase in CD44 cell surface expression (Figure 7A). Moreover, EMT-associated downregulation of E-cadherin and upregulation of fibronectin and vimentin, both at the protein and mRNA level, were prevented by anti-TGF-β1 antibody treatment (Figure 7B-7D). Taken together, these data suggest that a consequence of decreased β2 subunit proteasome activity in HMLE cells is the stabilization of the TGFR2 at the cell surface, thereby resulting in increased TGF-β1 signaling and induction of the EMT transcriptional program.

## DISCUSSION

It is established that EMT can generate cells with CSC properties, but the molecular mechanisms that induce EMT have not been fully elucidated. In the present study, we found that epithelial cells decrease their proteasome activity during EMT and that proteasome inhibitors can induce EMT. Mechanistically, we show that one consequence of downregulated proteasome activity may be increased TGF-β1 signaling, a potent inducer of EMT. However, it remains possible that a decrease in proteasome activity may enhance the stability of other EMT-promoting factors. For example, glycogen synthase kinase-3β (GSK3β)-mediated phosphorylation of SNAI1 has been shown to facilitate its nuclear export and degradation [9, 33], while SNAI2 can undergo proteasome-dependent degradation mediated by the p53-MDM2 complex [9, 34]. In addition, phosphorylation of TWIST1 by the MAPK p38-JNK-ERK complex protects it from degradation [9, 35]. Thus, downregulation of proteasome activity may lead to the induction of the EMT program through effects mediated by other pathways in addition to enhanced TGF-β1 signaling.

In support of the potential clinical relevance of our observations, we show that proteasome subunit mRNA expression in human tumor samples correlates with disease progression (Figure 4A, 4B). These results are consistent with the finding that low expression of 19S proteasome subunit *PSMD1* correlates with decreased probability of overall survival in a cohort of 82 head and neck squamous cell carcinoma patients [11]. Conversely, high expression of proteasome subunits *PSMB7* [36] and *PSMB4* [37] has been shown to be associated with decreased breast cancer patient survival and poor prognosis, respectively. The extent to which the expression of *PSMD1*, *PSMB4*, and *PSMB7* affect proteasome activity remains poorly understood, and may provide a possible explanation for these contrasting observations. To our knowledge, ours is the first study that suggests a correlation between decreased expression of the catalytic proteasome subunits - *PSMB2* and *PSMB5* - and reduced survival of breast cancer patients.

Finally, our findings are intriguing in light of the clinical use of proteasome inhibitors for the treatment of cancer. While many patients with hematopoietic malignancies respond to the proteasome inhibitor bortezomib, clinical trials investigating the use of proteasome inhibitors for solid tumors have thus far been disappointing [38-40]. Our data suggest a possible molecular mechanism for this observed effect - that pharmacologic inhibition of the proteasome may result in induction of EMT and acquisition of certain attributes of CSCs. Paradoxically, our results also suggest the possibility that pharmacologic inhibition of the proteasome may not only induce EMT in breast cancer cells, but may also endow them with an enhanced capacity to survive against the stimuli that led to their induction in the first place (Figure 2E). Taken together, these results suggest caution in the use of proteasome inhibitors in tumor subtypes that follow the CSC paradigm and raise the possibility that the use of agents that activate the proteasome, such as inhibitors of the deubiquitinase USP14 [41], might instead be an effective therapeutic strategy in such cancers.

## MATERIALS AND METHODS

### Mice

All animal work was approved by the Institutional Animal Care and Use Guidelines of the University of California, San Diego. All mice were housed in specific pathogen-free conditions prior to use.

### Cell culture

Immortalized human mammary epithelial cells (HMLE), HMLE-Snail, HMLE-Twist, HMLE-Ras (HMLER), and HMLER-Twist were maintained as previously described [32, 42]. MCF10A cells were obtained from Dr. Karra Muller (UCSD) and cultured in Dulbecco's Modified Eagle's Medium/Nutrient Mixture F-12 supplemented with 5% FBS, 20ng/ml EGF, 0.5mg/ml Hydrocortisone, 10ug/ml Insulin, 100ng/ml Cholera toxin, and Penicillin-Streptomycin. TGF-β1 treatment was performed as previously reported [6]. Epoxomicin (Enzo Life Sciences) was used at 12.5μM. Anti-TGF-β1 neutralizing antibodies (Bio X Cell) were used at a concentration of 10μg/mL.

### Activity-based proteasome probes and proteasome inhibitors

Subunit-selective activity-based proteasome probes, MVB003 (pan-reactive), LW124 (β1 subunit-reactive), and PR592 (β5 subunit-reactive) and subunit-selective proteasome inhibitors, NC001 (β1 subunit-reactive), LU102 (β2 subunit-reactive), and LU005 (β5 subunit-reactive) were reconstituted in DMSO and have been previously described (Supplementary Figure S1) [24-27]. In brief, the proteasome inhibitors were designed for selective and irreversible binding to proteolytically active proteasome subunits. Activity-based probes (ABPs) are similar to the proteasome inhibitors, except that they carry a fluorescent group that allows visualization of proteins bands by SDS-PAGE. For selective proteasome inhibition, cells were cultured in media containing 5% fetal bovine serum in the presence of 5μM β1 inhibitor, 5μM β2 inhibitor, or 0.5μM β5 inhibitor up to 14 days. For analysis of short-term effects of proteasome inhibition on TGF-β signaling, cells were treated for 24 hours.

### In-gel proteasome activity assay

Cells were mechanically disrupted in 50mM Tris, pH7.4. 100 μg of total protein was incubated with 0.5μM pan-reactive probe (MVB003), 0.5μM β1 probe (LW124), or 1μM β5 probe (PR592) for 3 hrs at 37°C in 50mM Tris, pH7.4 buffer supplemented with 5mM MgCl_2_, 250mM sucrose, 1mM DTT, and 2mM ATP. After the reaction, samples were resolved on Novex 4-20% Tris-Glycine Mini Protein Gels (Life Technologies) and the fluorescent signals were detected using FluorChem Q (ProteinSimple). Densitometry was used to quantify the signals. Because the activity-based probes bind stoichiometrically to selective proteasome subunits, the fluorescent signal measured is linearly proportional to the activity of the specific β subunit. Beta2 subunit activity was determined by using the pan-reactive probe (MVB003) and analyzing the the fluorescent band at ∼23 kDa.

### Immunoblotting and antibodies

Cells were lysed on ice in buffer containing 50mM HEPES, pH7.4, 80mM NaCl, 5mM MgCl_2_, 10mM EDTA, 5mM sodium pyrophosphate*10H_2_O, 1% TritonX-100, and Protease Inhibitor Cocktail (Sigma-Aldrich). 30 μg of total protein from each sample was resolved on Novex 4-20% Tris-Glycine Mini Protein Gels and transferred onto nitrocellulose membranes. The blots were probed with the appropriate antibodies: anti-ubiquitin (Cell Signaling Technology), anti-β-actin (Sigma-Aldrich), anti-20S/β1 subunit (Santa Cruz Biotechnology, Inc.), anti-20S/β2 subunit (Santa Cruz Biotechnology, Inc.), anti-20S/β5 subunit (Santa Cruz Biotechnology, Inc.), anti-E-cadherin (BD Biosciences), anti-fibronectin (BD Biosciences), anti-vimentin (Cell Signaling Technology), anti-phospho-Smad2 (Ser465/467) (Cell Signaling Technology), or anti-Smad2/3 (Cell Signaling Technology). Signals were detected using Odyssey infrared imaging system (LI-Cor Biosciences).

### Flow cytometry analysis and cell sorting

Suspensions of HMLE or MCF10A cells were stained with anti-CD44 antibody (Biolegend), anti-CD24 antibody (Biolegend), 7-AAD (eBioscience), Annexin-V (eBioscience), or anti-TGFR2 antibody (R&D Systems) and analyzed by flow cytometry on a BD Accuri C6 (BD Biosciences). Data were analyzed using FlowJo software. Cell sorting was done using at FACSAria (BD Biosciences) at the UCSD Human Embryonic Stem Cell Core Facility.

### Mammosphere culture

Mammosphere culture was performed as previously described [6, 43], except that 2000 single cells for the primary culture and 1000 dissociated cells for the serial passages were plated per well of a 24-well plate. For serial passage studies, mammospheres were first dissociated into single cells and then plated in mammosphere culture conditions. This process was repeated three times.

### Tumorigenesis assay

Five million HMLER cells per group (control HMLER, HMLER-Twist, or HMLER+β2 inhibitor) were injected subcutaneously into homozygous nude mice. A total of 12 animals were used for each condition. The tumor incidence and the tumor size were monitored for two months following injection.

### Confocal microscopy

Following growth on coverslips for 2 days and treatment with DMSO, β2 inhibitor, β5 inhibitor or TGF-β1 with or without anti-TGF-β1 antibodies, immunofluorescence of HMLEs and MCF10A was performed as previously described [44] using anti-E-cadherin (BD Biosciences), anti-fibronectin (BD Biosciences), anti-vimentin (Cell Signaling Technology), or anti-Smad4 (B-8) (Santa Cruz Biotechnology) followed by anti-rabbit Alexa Fluor 555 or anti-mouse Alexa Fluor 488 (Life Technologies) antibodies. DAPI (Life Technologies) was used to detect DNA. Acquisition of image stacks was performed as previously described [44] using a FV1000 laser scanning confocal microscope (Olympus). Fluorescence within the nucleus or cytoplasm was quantified using ImageJ software.

### Microarray analysis

Total RNA was isolated from HMLE cells using TRIzol (Life Technologies). RNA was extracted from TRIzol using chloroform and precipitated with isopropanol. cDNA was then synthesized using the High Capacity cDNA Reverse Transcription Kit (Life Technologies) and hybridized to HumanHT-12_v4 arrays according to standard protocols (Illumina). Raw data were quantile normalized.

Differential expression analysis of Affymetrix array data was conducted using the Array Studio analysis suite (Omicsoft, Inc). Datasets were quantile-normalized at the gene-level and log-transformed before computing one-way ANOVA statistics between treatment and control group samples. Principal component analysis was used to detect possible outliers and batch effects across samples. Differentially expressed gene signatures representing 1-5% of the coding transcriptomes were generated using a FDR < 0.0001. Functional enrichment of the differentially expressed genes was performed using GSEA, as previously described [45, 46], and IPA (Ingenuity). Genes significantly upregulated and downregulated in HMLE-Snail were used as the enrichment set. Other enrichment sets used were taken from the Molecular Signatures Database [45]. Enrichment set names are included in Supplementary Table 1.

### Real-time quantitative PCR

Total RNA was extracted using TRIzol (Life Technologies) and was reverse-transcribed with MultiScribe Reverse Transcriptase (Life Technologies). The resulting cDNAs were used for qPCR using SsoAdvance SYBR Green Supermix (Bio-Rad) in triplicate. PCR and data collection were performed on CFX96 Touch Real-Time PCR Detection System (Bio-Rad). All the values were normalized to an internal control GAPDH. Relative expression for each target gene was compared to that of HMLE or MCF10A, and the data were presented as relative fold change. See Supplementary Information for primer sequences.

### Kaplan-Meier analysis

Kaplan-Meier survival curves were generated from The Cancer Genome Atlas Breast Invasive Carcinoma Illumina HiSeq gene expression dataset (*n* = 1215). Patients were stratified based on the combined expression of proteasome catalytic subunit genes *PSMB2* and *PSMB5*. High and low expression is defined as the top 20% (*n* = 245 of 1215) and bottom 22% (*n* = 277 of 1215) of the patient distribution, respectively. Expression and overall survival data were downloaded from the UCSC Cancer Genomics Browser (https://genome-cancer.ucsc.edu/). Prism (GraphPad) was used to test the statistical significance between the ­overall survival curves using the Log-rank (Mantel-Cox) Test.

### Oncomine analysis

Oncomine Platform (v4.5, Life Technologies) was used to analyze single gene expression in normal *versus* tumor samples, using the Finak Breast dataset [47].

### Statistics

Statistical analyses were performed with a unpaired *t* test using GraphPad Software. The resulting statistics are indicated in each figure as follows: ns = Not significant (*P* > 0.05), Significant: * = (*P* ≤ 0.05), ** = (*P* ≤ 0.01), *** = (*P* ≤ 0.001), **** = (*P* ≤ 0.0001).

## SUPPLEMENTARY MATERIAL FIGURES AND TABLE


