# Supplementary material for: Downregulation of 26S proteasome catalytic activity promotes epithelial-mesenchymal transition
Source: Oncotarget. 2016 Feb 22;7(16):21527–41. doi: 10.18632/oncotarget.7596 (PMC5008303; doi:10.18632/oncotarget.7596)
Supplement: Supplementary file 1 [file oncotarget-07-21527-s001.pdf]

Downregulation of 26S proteasome catalytic activity promotes epithelial-mesenchymal transition

Supplementary Material

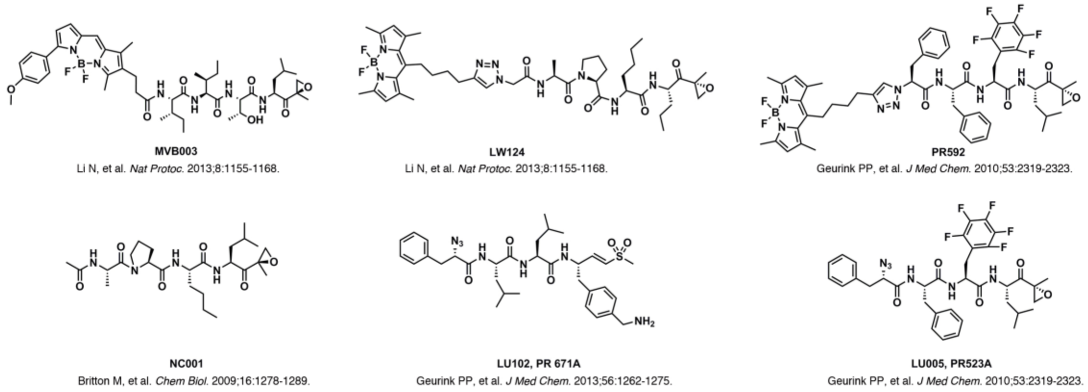

Supplementary Figure S1. Structures of proteasome activity-based probes and selective proteasome inhibitors. The pan-reactive (MVB003), β1 (LW124), β5 (PR592) subunit-specific probes, and β1 (NC001), β2 (LU102, PR671A), β5 (LU005, PR523A) subunit-specific inhibitors used in the study have been previously described (23-26).

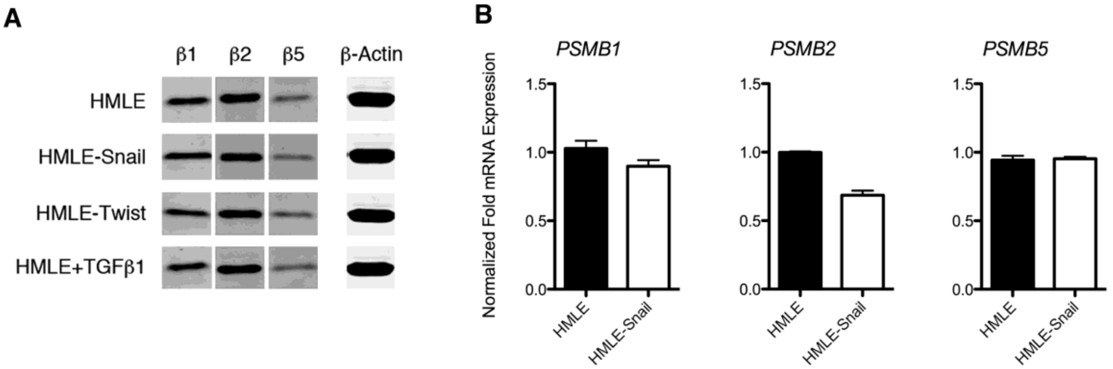

Supplementary Figure S2. Proteasome subunit expression is unchanged during EMT. (**A**) Total protein expression of the β1, β2, and β5 catalytic subunits was assessed by Western blotting with respective antibodies. A representative blot is shown. The blot was also probed for β-Actin, as a loading control. The vertical space inserted between lanes indicates where we removed intervening, irrelevant samples. (**B**) mRNA levels of HMLE parental cells and HMLE cells overexpressing Snail (HMLE-Snail). Data are shown as fold-change normalized to HMLE. Error bars indicate SEM (n = 3).

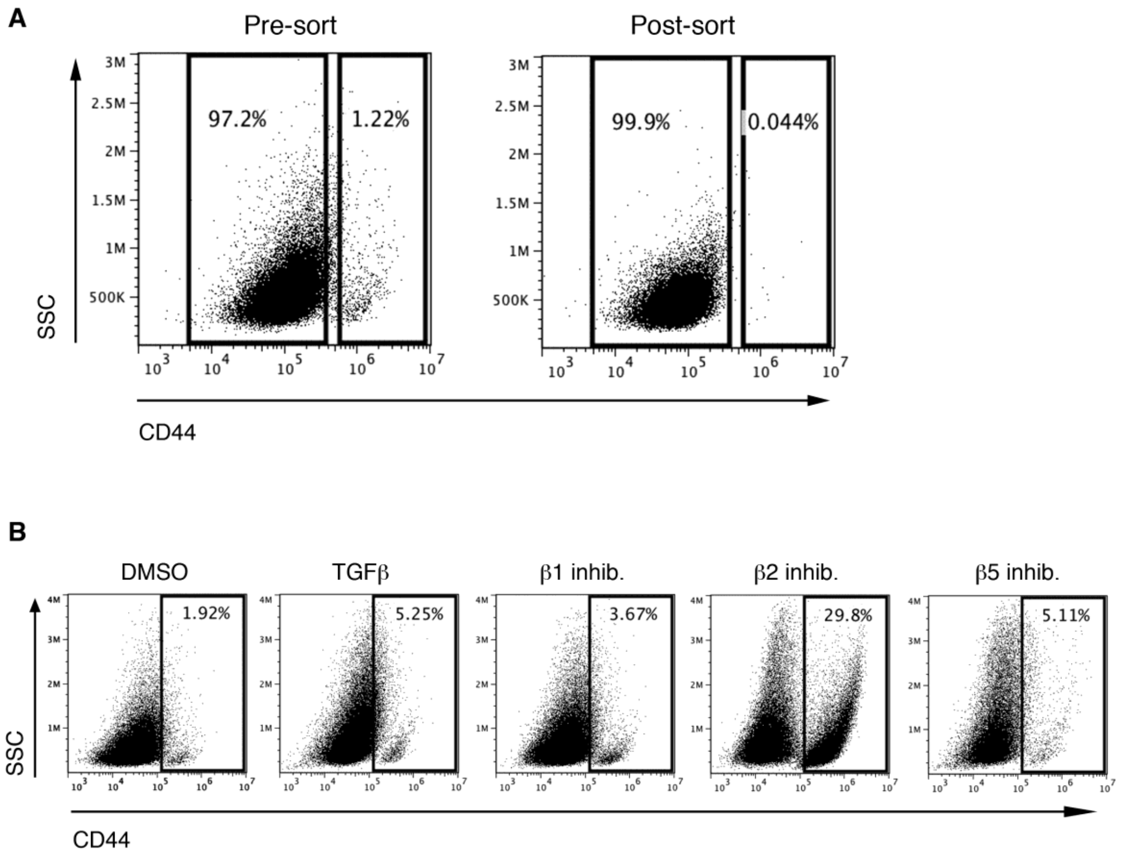

Supplementary Figure S3. HMLE CD44hi cells arise from CD44low cells during EMT or proteasome inhibitor treatment. (**A**) Flow cytometry analysis of CD44 surface expression and side scatter (SSC) of HMLE parental cells before (“Pre-sort”) and after (“Post-sort”) sorting for low expression of CD44. Percentage of CD44low cells (left gate) and CD44hi cells (right gate) within the live population is indicated. (**B**) Flow cytometry analysis of CD44 surface expression and side scatter (SSC) of HMLE CD44low cells treated with DMSO, TGF-β, β1 inhibitor, β2 inhibitor, or β5 inhibitor for 14 days. Percentage of CD44hi cells within the live population is indicated.

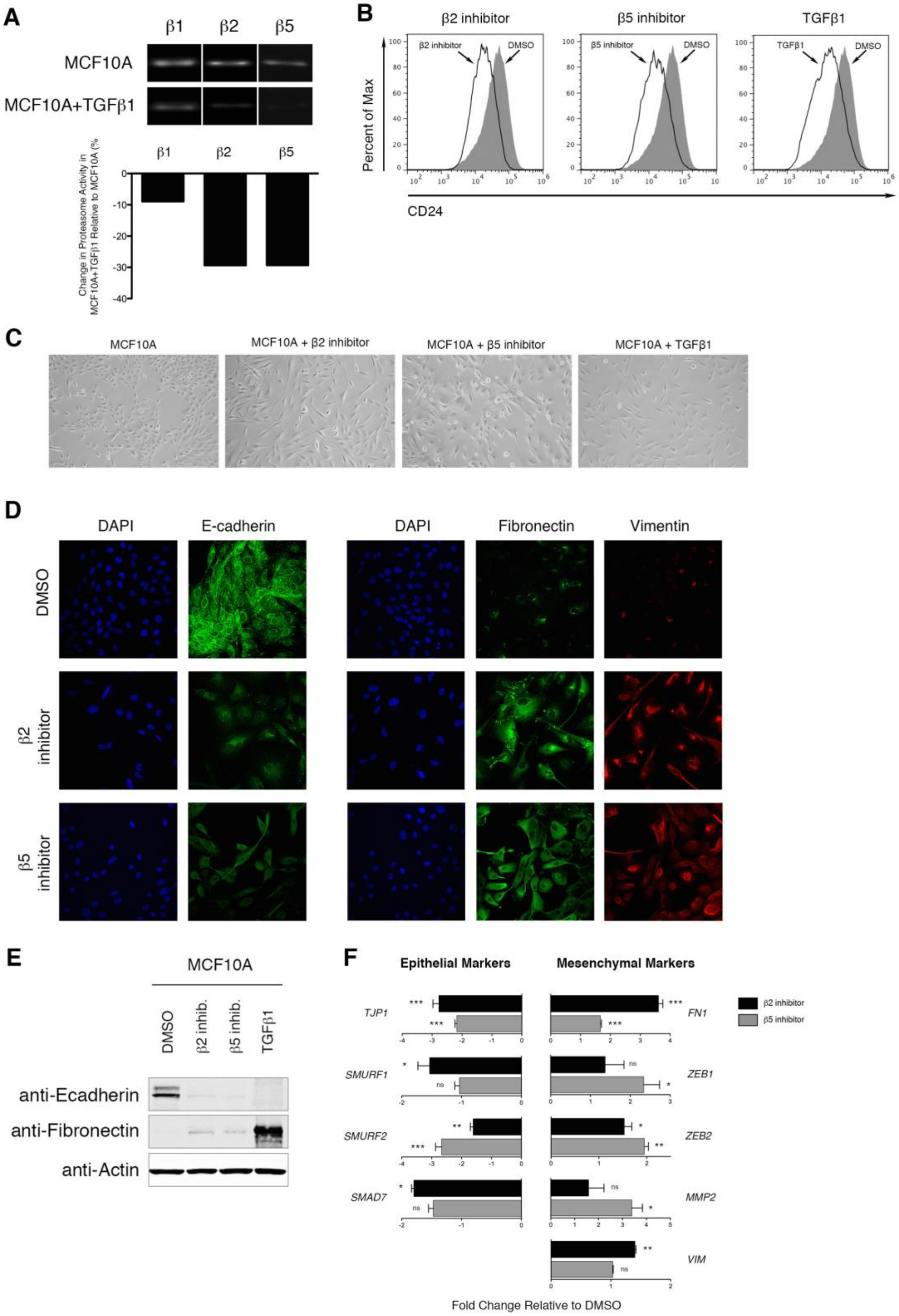

Supplementary Figure S4. Downregulation of proteasome activity is associated with EMT in MCF10A cells. (**A**) β1, β2, and β5 subunit proteasome activity in MCF10A

alone or treated with TGF-β1 was measured by in-gel proteasome activity assay. A representative SDS-PAGE gel is shown. Vertical spaces inserted between lanes indicate removal of intervening, irrelevant samples. All the samples were run on the same gel and imaged in a single scan. Quantification of β1, β2, and β5 subunit activity is presented as percent change relative to MCF10A. (**B**) Flow cytometry analysis of CD24 surface expression in MCF10A after approximately 14 days of treatment with β2 subunit inhibitor, β5 subunit inhibitor, or TGF-β1 (open histograms) compared to DMSO treatment (grey-shaded histogram). (**C**) Representative brightfield images of MCF10A treated with DMSO or β2 inhibitor, β5 inhibitor, and TGF-β1 after 14 days of treatment. All the images were taken at 10X magnification. (**D**) Confocal microscopy of E-cadherin (left panel; green), Fibronectin (right panel; green), or Vimentin (red) in MCF10A cells treated with β2 subunit inhibitor or β5 subunit inhibitor. Images were taken at 40X magnification. (**E**) Immunoblot of whole cell lysates from MCF10A cells treated with DMSO, β2 inhibitor, β5 inhibitor, or TGF-β1 using anti-E-cadherin, and anti-Fibronectin antibodies, representative of 3 independent experiments. β-Actin served as a loading control. (**F**) mRNA levels of epithelial (*TJP1*, *SMURF1*, *SMURF2*, and *SMAD7*) and mesenchymal markers (*FN1*, *ZEB1*, *ZEB2*, MMP2, and *VIM*) in MCF10A+β2 inhibitor (black bars) or MCF10A+β5 inhibitor (grey bars). GAPDH was used as a reference gene. Data are shown as fold-change relative to DMSO-treated MCF10A. Error bars indicate SEM ($n \geq 3$).

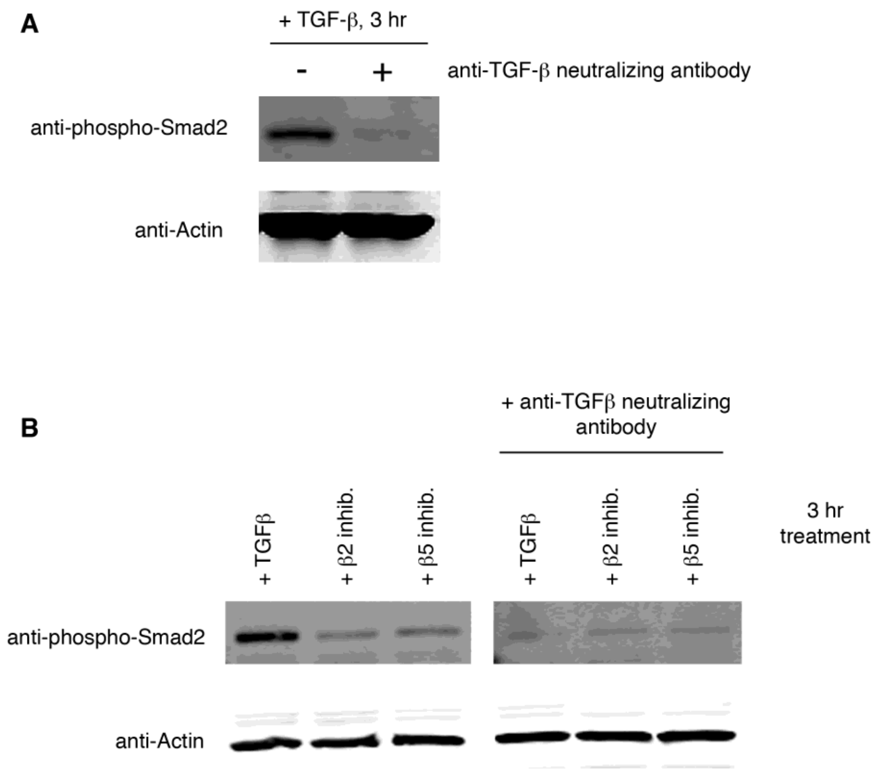

Supplementary Figure S5. Anti-TGF-β neutralizing antibody functions to block TGF-β signaling. (**A**) Immunoblot of whole cell lysates from HMLE cells treated with 2.5 ng/ml TGF-β for 3 hours with or without 10 μg/ml anti-TGF-β neutralizing antibody. (**B**) Immunoblot of whole cell lysates from HMLE cells treated with 2.5 ng/ml TGF-β or β2 or β5 proteasome subunit inhibitors for 3 hours, with or without 10 μg/ml anti-TGF-β neutralizing antibody.

Supplementary Table 1

Category	Gene Set Name	Size	β2 vs DMSO NES	β2 vs DMSO Nom. p-value	β2 vs DMSO FDR	β5 vs DMSO NES	β5 vs DMSO Nom. P-value	β5 vs DMSO FDR
Epithelial Characteristics	PROTEINACEOUS_EXTRACELLULAR_MATRIX	98	-1.637	0.002	0.022	-1.723	0.000	0.020
	BASOLATERAL_PLASMA_MEMBRANE	34	-1.615	0.017	0.016	-1.533	0.019	0.030
Epithelial-Mesenchymal Transition	SARRIO_EPITHELIAL_MESENCHYMAL_TRANSITION_UP	165	2.804	0.000	0.000	2.839	0.000	0.000
	ZHANG_BREAST_CANCER_PROGENITORS_UP	233	2.106	0.000	0.000	2.302	0.000	0.000
	ALONSO_METASTASIS_EMT_UP	36	2.073	0.000	0.000	1.868	0.002	0.000
	AIGNER_ZEB1_TARGETS	35	1.655	0.006	0.004	1.714	0.000	0.001
	WANG_TUMOR_INVASIVENESS_UP	244	1.650	0.000	0.003	1.808	0.000	0.000
Metastasis	WINNEPENNINCKX_MELANOMA_METASTASIS_UP	148	2.539	0.000	0.000	2.668	0.000	0.000
	ALONSO_METASTASIS_UP	188	2.199	0.000	0.000	2.111	0.000	0.000
	WANG_METASTASIS_OF_BREAST_CANCER_ESR1_UP	20	1.718	0.004	0.012	1.935	0.000	0.000
	TOMIDA_METASTASIS_UP	26	1.491	0.050	0.094	1.471	0.045	0.036
	CROMER_METASTASIS_UP	75	1.341	0.040	0.118	1.338	0.049	0.078
	RICKMAN_METASTASIS_UP	227	1.203	0.079	0.191	1.153	0.127	0.225
TGFβ	KARAKAS_TGFB1_SIGNALING	18	1.735	0.002	0.007	1.779	0.004	0.013
	COULOUARN_TEMPORAL_TGFB1_SIGNATURE_UP	103	1.578	0.004	0.057	1.752	0.000	0.009
	JAZAG_TGFB1_SIGNALING_UP	104	1.455	0.008	0.107	1.458	0.010	0.092
	RECEPTOR_BINDING	252	1.274	0.033	0.194	1.415	0.008	0.099

Supplementary Table 1. GSEA with curated gene sets from the Molecular Signatures Database. Gene sets were selected that describe phenotypes and processes related to EMT. β2 and β5 inhibitor-treated HMLE cells are positively enriched in EMT, metastasis, and TGF-β-related gene sets, while negatively enriched in epithelial gene sets. Size indicates the number of genes within Gene Set. NES, Normalized Enrichment Score. Nom., Nominal. FDR, false discovery rate.

Supplementary Methods

Real-Time Quantitative PCR

The following primers were used:

hCDH1: 5’-TGCCCAGAAAATGAAAAAGG

hCDH1: 3’-GTGTATGTGGCAATGCGTTC

hTJP1: 5’-GTCTGCCATTACACGGTCCT

hTJP1: 3’-GGTCTCTGCTGGCTTGTTTC

hCLDN1: 5’-GTGGAGGATTTACTCCTATGCCG

hCLDN1: 3’-ATCAAGGCACGGGTTGCTT

hSMURF1: 5’-TGTGAAAAACACATTGGACCCA

hSMURF1: 3’-ACGCTAATGGTTATCGAATCCG

hSMURF2: 5’-TATGCAAACTCGGGCCAAATG

hSMURF2: 3’-CCTGTGCCTATTCGGTCTCTG

hSMAD7: 5’-GGACGCTGTTGGTACACAAG

hSMAD7: 3’-GCTGCATAAACTCGTGGTCATTG

hCDH2: 5’-ACAGTGGCCACCTACAAAGG

hCDH2: 3’-CCGAGATGGGGTTGATAATG

hSNAI1: 5’-CCTCCCTGTCAGATGAGGAC

hSNAI1: 3’-CCAGGCTGAGGTATTCCTTG

hZEB2: 5’-TTCCTGGGCTACGACCATAC

hZEB2: 3’-TGTGCTCCATCAAGCAATTC

hZEB1: 5’-CCTGTCCATATTGTGATAGAGGC

hZEB1: 3’-ACCCAGACTGCGTCACATGT

hFOXC2: 5’-GCCTAAGGACCTGGTGAAGC

hFOXC2: 3’-TTGACGAAGCACTCGTTGAG

hTWIST1: 5’-GGAGTCCGCAGTCTTACGAG

hTWIST1: 3’-TCTGGAGGACCTGGTAGAGG

hMMP2: 5’-TGCCTGGAATGCCAT

hMMP2: 3’-GTTCTCCAGCTTCAGGTAAT

hMMP9: 5’-AGCTCATGGGGACTCCTACC

hMMP9: 3’-AGACTGCTACCATCCGTCCA

hGAPDH: 5’-ACCCAGAAGACTGTGGATGG

hGAPDH: 3’-TCTAGACGGCAGGTCAGGTC

hSIP1: 5’-TTCCTGGGCTACGACCATAC

hSIP1: 3’-TGTGCTCCATCAAGCAATTC

hVIM: 5’-GAGAACTTTGCCGTTGAAGC

hVIM: 3’-GCTTCCTGTAGGTGGCAATC

hFN1: 5’-CAGTGGGAGACCTCGAGAAG

hFN1: 3’-TCCCTCGGAACATCAGAAAC
